# Enhancing Single Molecule Imaging in Optofluidics and Microfluidics

**DOI:** 10.3390/ijms12085135

**Published:** 2011-08-12

**Authors:** Andreas E. Vasdekis, Gregoire P.J. Laporte

**Affiliations:** Optics Laboratory, School of Engineering, Ecole Polytechnique Fédérale de Lausanne (EPFL), Lausanne CH-1015, Switzerland; E-Mail: gregoire.laporte@epfl.ch

**Keywords:** optofluidics, microfluidics, single molecule, fluorescence, imaging, surface passivation, micro-fabrication, lab-on-a-chip

## Abstract

Microfluidics and optofluidics have revolutionized high-throughput analysis and chemical synthesis over the past decade. Single molecule imaging has witnessed similar growth, due to its capacity to reveal heterogeneities at high spatial and temporal resolutions. However, both resolution types are dependent on the signal to noise ratio (SNR) of the image. In this paper, we review how the SNR can be enhanced in optofluidics and microfluidics. Starting with optofluidics, we outline integrated photonic structures that increase the signal emitted by single chromophores and minimize the excitation volume. Turning then to microfluidics, we review the compatible functionalization strategies that reduce noise stemming from non-specific interactions and architectures that minimize bleaching and blinking.

## 1. Introduction

Since the first attempts at low temperature [1,2], near [3] or far field [4–7] single molecule imaging has evolved into a very powerful method of unmasking dynamic heterogeneities of complex material [8] or biological systems [9,10]. In the majority of cases, single molecule imaging takes place in fluorescence; in this imaging modality, light at a specific wavelength is absorbed by the molecule, which in turn emits a Stokes-shifted signal. This signal is collected via high numerical-aperture optics and projected onto a sensitive imaging charge coupled device sensor (CCD), thus enabling its localization. It is worth noting that imaging is part of the much broader field of single molecule detection (SMD), where techniques such as Fluorescence Correlation Spectroscopy (FCS), spectroscopy and general optical sensing enable the detection of the presence and the activity of single molecules but not necessarily their localization [11].

Single molecule imaging has been particularly aided by the possibility of trangsenically inducing light emission capabilities in living organisms [12–14], but also by more recent efforts of sequencing [15–17] and superresolution. Superresolution can be based on spectral multiplexing [18], digitization for localization [19,20], singlet state population manipulation (STED) [21], and time multiplexing via polarization [22], or photoactivation [23–26]. Apart from recent efforts in probing single fluorescent proteins in solution using electrokinetic traps [27] and more traditional ones like FCS [28], the vast majority of single molecule imaging studies take place on surfaces. Microfluidic systems are synergetic to this detection principle as they are compatible with many anchoring chemistries, but simultaneously enable variable delivery of bioentities, thus achieving unsurpassed levels of multiplexing [29,30].

Microfluidics manipulates liquids at sub-millimeter length scales, enabling reduced reagent consumption and highly multiplexed studies by lithographically defining microfluidic channels in close proximity to each other. To this end, it has attracted substantial attention the past years in fundamental studies [31], as well as a wide range of applications, including microscale analysis systems [32–36], information processing [37,38], bioentity manipulation [39–41], and chemical synthesis [42]. Common materials for fabricating microfluidics are glass [35,43] and elastomeric polymers, such as the poly(dimethylsiloxane) (PDMS) [44]. While both materials are transparent in the visible range, PDMS exhibits relatively more advantages as it is more cost-effective, compatible with replica-molding, and its elastomeric nature enables flow control and object manipulation [45].

More recently, optofluidics emerged as the fusion of integrated optics with microfluidics [46,47], aiming primarily at novel photonic structures, such as light sources [48], waveguides [28] and optical modulators [49], as well as analytical methods, such as mass transport [50], on-chip imaging [51] and molecular manipulation [52]. More relevant to the present work is the recent report on surface optofluidics, where surfaces undertake an optical or chemical character to enable or enhance optofluidic functions [53].

**Figure 1.**
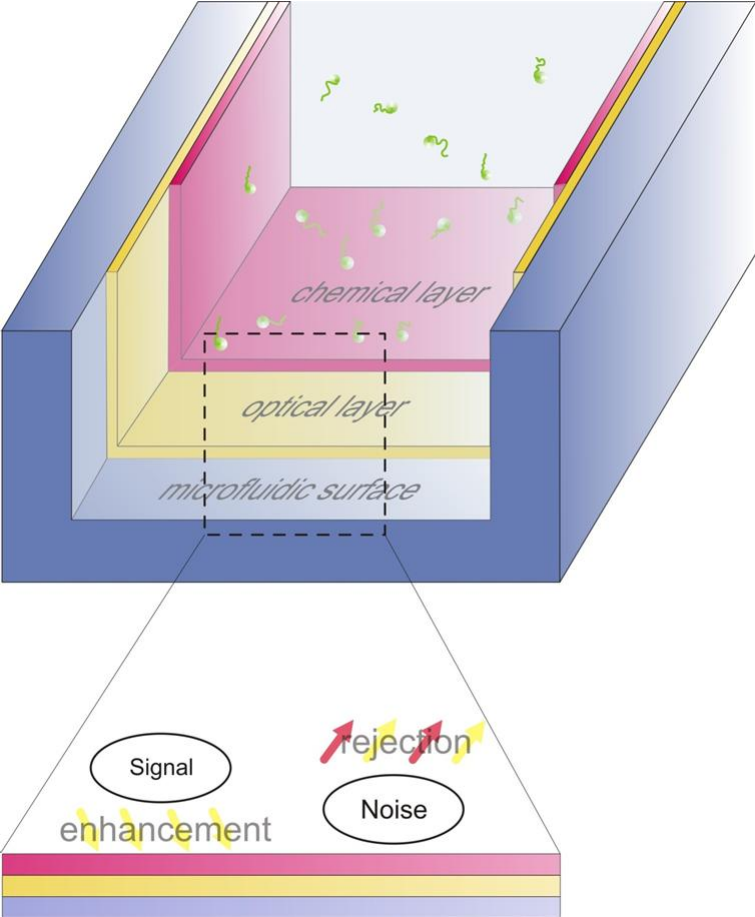
**Upper:** a general schematic illustrating the aim of this work: we review the optical and chemical layers compatible with conventional microfluidics that enhance the signal to noise ratio during single molecule imaging. **Lower:** This enhancement is achieved by decreasing the noise stemming from non-specific interaction (chemical layer), and by enhancing the signal via modifications of the electromagnetic and electrostatic environment of the single emitter (optical layer).

Within this paper, we review recent reports on enhancing the signal to noise ratio during single molecule imaging in optofluidics and microfluidics (Figure 1). One way to achieve this type of enhancement is by increasing the signal emitted by single chromophores that reach the detector. We will review such methods in the optofluidic section (Section 2), highlighting the photonic structures that can be integrated with microfluidic channels. Similar performance enhancement can be achieved by minimizing the signal detected due to the presence of un-wanted molecules in the observation volume. To address this, there are in general two approaches. The first approach relates to the reduction of the observation volume, which can be achieved by both optofluidics (Section 2) and chemical techniques (Section 3). The second approach relates to the reduction of non-specific interactions, or equivalently the minimization of the number of un-wanted molecules within the observation volume. This is reviewed in section 4, where the related chemical strategies compatible with microfluidics are discussed.

## 2. Signal to Noise Ratio Enhancement in Optofluidics

Multiple methods have been developed to push the fluorescence detection by employing photonic structures [54–56]. Such photonic structures exhibit a certain resonance frequency bandwidth; within this bandwidth they can passively either amplify light and/or modify its radiation pattern. Light in both cases can be either incident on such structures or be internally generated within them. In this paper, we will focus on the integration of such photonic structures with microfluidics. Such optofluidic examples involve the ‘zero mode waveguide’ [57], integrated ARROW waveguides for FCS [58], and evanescent wave resonators [52,59,60]. We will highlight however only optofluidic methods for single molecule imaging and localization; such optofluidic principles are mostly related to one or a combination of the following:

1. Confinement of the excitation volume to reduce the background;
2. Confinement of the excitation density to enhance the pumping rate;
3. Modification of the radiative and non-radiative rates of the chromophores;
4. Scattering enhancement and optical loss minimization to increase the signal reaching the detector;
5. Modification of the emission pattern in order to improve the collection efficiency.

**Figure 2.**
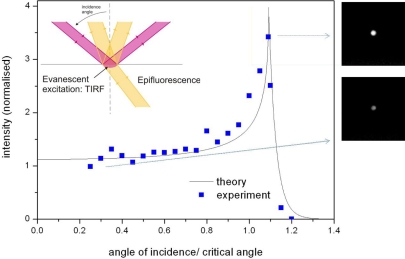
Characteristic intensity enhancement around the critical angle at the interface (0.2 * wavelength) between a glass-coverslip and water (the wavelength is 488 nm). The experimental curve is the integrated signal of fluorescently labeled polystyrene beads (diameter of 0.8 μm) adsorbed on the coverslip surface and coated with a water droplet; the theoretical one is the evanescent wave intensity at the water-glass interface, calculated from [67]. The inset illustrates the geometries of epifluorescence and total internal reflection.

Total internal reflection microscopy (TIRF) is one of the most conventional methods for fluorescence signal enhancement and, due to its compatibility with microfluidics, will be reviewed first for completeness, followed by methods to enhance the fluorescence emission by either employing metallic or dielectric micro- and nanostructures. In TIRF, evanescent waves are generated when light is totally reflected at the interface between two dielectric media (inset of Figure 2). The evanescent wave decays exponentially from the interface and thus selectively illuminates fluorophores in the close proximity to the interface (100’s nm) [61,62]. The most common TIRF configurations use a prism [55,63], or a high NA objective [64,65]. The excitation intensity and the resulting fluorescent signal [61,66] are several times stronger in the TIRF condition than in epifluorescence (Figure 2). This enhancement can be estimated by using the Fresnel equations [64,67,68] and is synergetic to the coupling to surface modes, known as the Goos-Hänchen shift. In Figure 2, the intensity of the surface wave is plotted at different angles of incidence and is compared to an experimental measurement.

**Figure 3.**
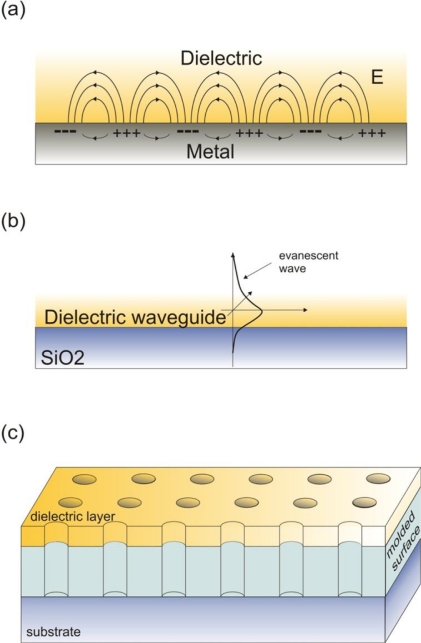
An illustration of the optofluidic technologies, highlighted in the text, namely (**a**) surface plasmon polaritons, which are surface waves propagating at a dielectric metal interface (+ and − stand for regions where the charge density is lower and higher, respectively); In (**b**), a basic dielectric waveguide is shown, comprising of a thin dielectric layer where the waves propagate; In (**c**), a schematic representation of a photonic crystal device is shown. The surface on the substrate is lithographically molded with the periodic structure and the thin dielectric layer is sputtered on the top.

An optofluidics-related approach to enhance the emission signal from single molecules is to employ surface plasmon polaritons (SPPs); these too are surface waves that only occur at the interface between a conductor and a dielectric [69] (Figure 3a). SPPs are excited when incident light interacts with the conductor’s free charges that in turn collectively oscillate in resonance with the light wave. SPPs propagate along the conductor/external medium interface and the capability to structure such interfaces at the nanometer scale can lead to their strong resonant localization, primarily stemming from constructive interference. Plasmonic fluorescence enhancement has been demonstrated in a wide range of architectures, such as nanoparticles [70–74], nanowires [75], thin films [76], lithographically defined single [77–79] or composite nanostructures [80–82]. There are many excellent reviews on the topic [83–86], so we shall only highlight the most recent advances and the advantages and disadvantages of this method.

The most common mechanisms involved in plasmonic fluorescence enhancement are higher scattering efficiency due to the metal presence, denser excitation localization and thus pumping rate, and increased radiative rate due to the change in the photonic environment of the isolated chromophore [72,87]. Most recently, an enhancement of 1340 was reported using bow-tie antennas [78]; these are lithographically defined plasmonic nanostructures with typical maximum dimensions in the range of 100’s nm with the capacity of strongly localizing the excitation field at their centre. Similar principles have recently found applications in non-linear imaging, by coating nanoparticles with a metal layer [88]. Typical disadvantages regarding plasmonic fluorescence enhancement are its inverse relationship to the emitter’s quantum efficiency, and the substantial quenching that occurs when the chromophore is closer to the metal surface [89,90]. Another disadvantage of plasmonic resonators is their small size, which in turn substantially reduces the field of view. In addition, the integration of metallic structures with PDMS microfluidics may suffer from inefficient sealing, requiring the use of a specifically patterned metal layer or coating with a patterned polymer layer [91] in order to eliminate the contact between PDMS and metallic surfaces.

Dielectric resonators coupled with single emitters have been also explored primarily within the context of quantum electrodynamics (QED) [92,93]. Similar principles have been applied to fluorescent imaging, albeit in the weak-coupling regime. One such dielectric architecture is thin films integrated immediately below the surface of interest (Figure 3b). One such embodiment is employed in TIRF, where films with appropriate optical properties and dimensions are used to resonantly enhance the evanescent field of totally internally reflected incident light via coupling to waveguide modes supported by the film [94–98]. Thin films can also be directly employed as waveguides, forming an integrated version of TIRF [99,100]; in these schemes light is end-coupled to the waveguide so that its evanescent intensity excites the fluorophores in the waveguide’s vicinity. More recently, a thin film was employed as an antenna, enhancing the collection efficiency to approximately 100% [101]. In this case, the emitters are embedded in a thin polymer layer, sandwiched between a higher index medium layer (sapphire) and air. The high index medium forces the emitted light to refract at smaller angles, thereby enhancing the collection efficiency.

Periodic dielectric structures have also found applications in fluorescent enhancement. One such example is stacks of thin films, which exhibit very high Bragg reflectivity within a certain wavelength range. When single molecules are placed in the proximity of such structures and their emission spectrum overlaps with the mirror resonance, the spectrum and spontaneous emission rate can be substantially modified and enhanced, respectively [102,103]. Photonic crystals are another type of periodic dielectric structures that have been explored to the same end (Figure 3c). These are in most cases lithographically defined in high index dielectrics with minimal absorption in the wavelength range of interest. If carefully selected, their periodicity can impose a phase matching condition for both the excitation and emission wavelength [104–107]. The operational principle is based on the coupling of both the external pump and the fluorescence signal into Bloch wave modes; these modes are ‘leaky’ modes, manifested by their strong evanescent near fields on the surface of the photonic crystal, which is critical to enhancing the excitation and emission extraction. Very recently, annular Bragg resonators for focusing the excitation light have been demonstrated, exhibiting an enhancement factor of 20 [108]. Colloidal microspheres, have been also employed, initially within the context of QED [109]. Recently, sub-wavelength focusing was demonstrated by illuminating latex microspheres with Gaussian beams; this high excitation confinement resulted in a 5-fold enhancement of the single molecule fluorescence [110]. Another practical effect of colloidal microspheres is the improvement in extraction by redirecting the light emitted at large angles toward the normal to the surface [103]. More recently, the possibility to replace high-NA oil immersion objectives with colloidal particles was demonstrated [111]; the use of low-NA objective lenses enabled high temperature and high photostability single molecule imaging. We note that dielectrics can be integrated with PDMS microfluidics due to the presence of a native oxide layer on their surfaces or their direct compatibility with O_2_ plasma (e.g., polymers).

## 3. Microfluidic Architectures for Minimizing Photobleaching and the Excitation Volume

One advantage of single molecule imaging is the higher time-domain resolution in interrogating molecular dynamics than is possible in ensemble studies. However, this can be hampered by the photostability of singlet exciton emission, due to O_2_ and intersystem crossing mediated bleaching and blinking. The latter gives rise to stochastic fluctuations not linked to the biological behavior itself. These challenges have been addressed in microfluidics by mixing oxygen scavengers and triplet quenchers in the imaging buffer [112], which recently, under careful design, enabled the shortest observation duration [113]. An alternative approach integrating the imaging channels with ones continuously ventilated with nitrogen was recently demonstrated [114]. Deoxygenation was possible due to the porous nature of PDMS, thus enabling measurements in the absence of the aforementioned enzymes that can interfere with biological activity [114]. Another possible time-domain resolution limitation, especially in single molecule fluorescence energy transfer (smFRET), is the fluidic speed that can be relatively high especially during fluidic mixing. Sophisticated microfluidic architectures can address this by reducing the flow velocities immediately after mixing, thereby enabling longer optical interrogations [115].

**Figure 4.**
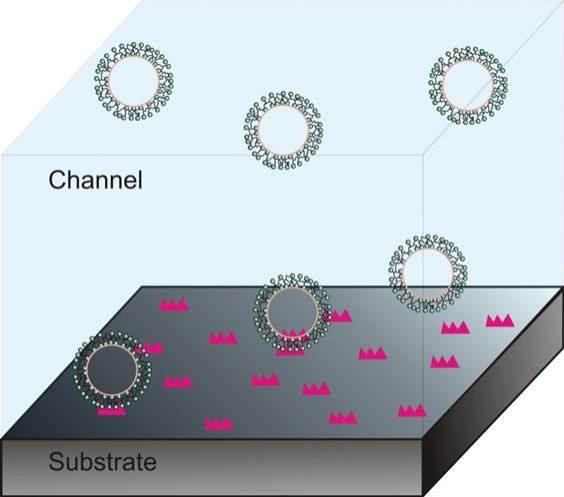
An illustration of vesicles tethered on a microfluidic surface and vesicles that freely float inside the bulk volume of the micro-channel.

Confocal imaging exhibits superior sensitivity by significantly restricting the excitation volume and thus minimizing the background fluorescence [4,116,117]. Microfluidic and nanofluidic systems can in principle achieve the same and push the limits of fluorescence detection. Within this paper, we will focus on the former, as many excellent reviews exist on the latter [118–120]. Apart from reduced size microfluidic channels employed in a recent report of multidimensional investigation of molecular energy landscapes and activities [30], volume restriction can be achieved by using micro-droplets [42,121]. The latter have not yet found widespread applications in single molecule imaging, contrary to vesicle encapsulation, which additionally exhibits much smaller volumes and the possibility of surface tethering [122–123]. In addition to the very small observation volumes, vesicle encapsulation (figure 4) enables the exclusion of any interaction of the contained molecules with the environment (including the surface), and as the possibility to combine surface immobilization with molecular recognition mechanisms, such as sequence specificity, thus forming a single molecule sensor of unlabeled oligonucleotides [124].

## 4. Noise Reduction Using Microfluidic Compatible Surface Passivation Strategies

Adhesion to biomaterials has been explored for many years, primarily focusing on the development of surfaces capable of non-specific protein adsorption for applications in implants, contact lenses and bioassays. Similar challenges have also been addressed in microfluidics. Microfluidic surfaces can act as anchoring points, where the molecules under investigation are immobilized. As discussed in the introduction, these attachment points need to exhibit certain properties in order to enhance the imaging of single molecules. In addition to their capacity to attract the ‘signal molecules’ with high affinity and resist non-specific adsorption of molecules that can contribute to noise [125–128], there are additional properties that we summarize below:

1. Simplicity and minimal preparation requirements: Single molecule imaging and detection is becoming a substantially interdisciplinary field, comprised of biologists, physicists and chemists. Hence, synergetic also to microfluidics, the chemical modification needs to be simple and require few implementation steps;
2. Compatibility with microfluidic fabrication: this necessitates that the surface chemistry is not compromised during fabrication, but also that the microfluidics retain their properties (e.g., mechanical) during surface treatment;
3. Bioactivity: The immobilized biomolecules must retain their activity, for example a protein that can unfold and refold, or a nucleic acid that can be recognized by site-specific proteins.

**Figure 5.**
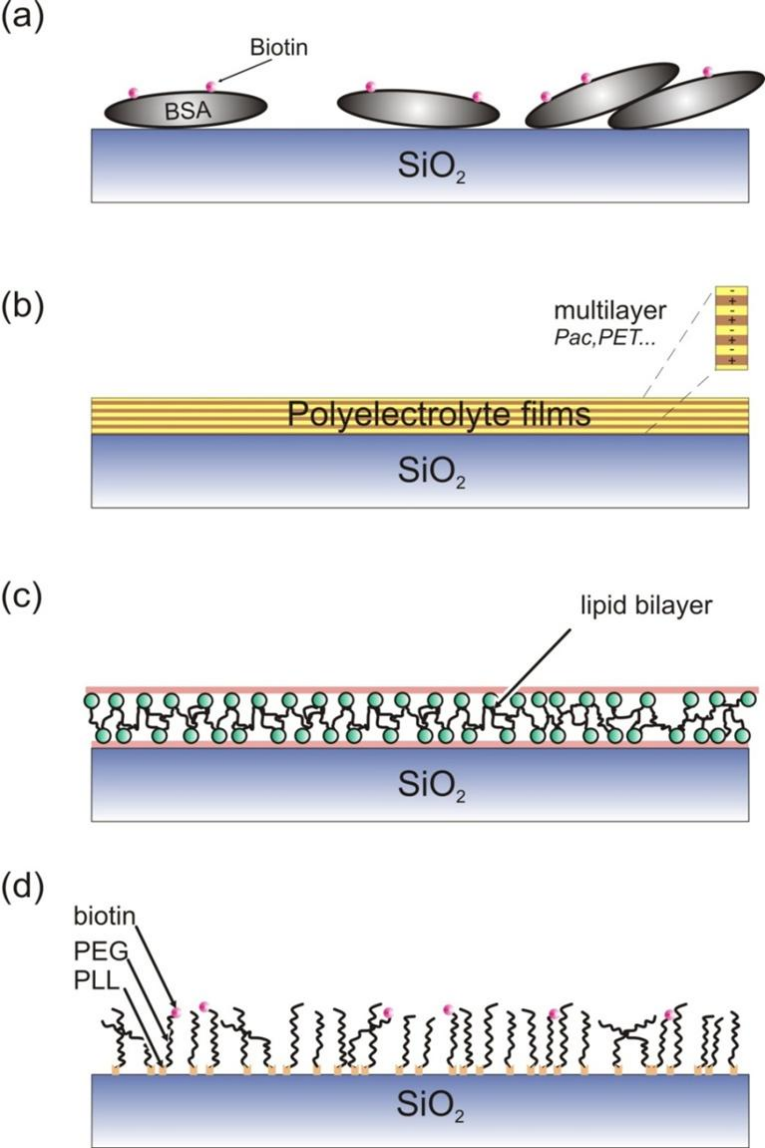
An illustration of some common surface passivation schemes in microfluidics: bovine albumin (BSA) adsorption (**a**), polyelectrolyte films (**b**), lipid bilayers (**c**) and PLL-*g*-poly(ethylene glycol) (PEG) copolymers.

Depending on the type of microfluidic devices and their fabrication, surfaces can be modified via a variety of chemistries. It is widely accepted that no single chemistry is compatible with multiple single molecule imaging analyses. Herein, we will not discuss gel confinement via polymerization [129,130] due to its contradiction to one of the main microfluidic advantages, namely that of flow-based delivery and multiplexing. On the contrary, we will focus on processes that can be performed either in-situ or ex-situ and comply with the aforementioned ‘ideal’ properties (Figure 5).

Self-assembly (SAM) and self-organization of molecular films have also been extensively reported. One area is the creation of monolayers of alkyl chains on thin gold films [131]. While such techniques are especially important in surface plasmon resonance measurements (SPR), more recently similar approaches have been proposed for PDMS, the cornerstone medium for microfluidics. In the latter, the PDMS pre-polymer is mixed with small molecules and then cast against a template with a specific surface energy [132,133]. During cross-linking, the small molecules in the PDMS solution are driven to the surface in order to match the energy of the mold surface. This method is particularly pertinent for the creation of chemical patterns, however to the authors’ knowledge the bonding with micro-channels is rather challenging to achieve using such surface treated templates.

**Figure 6.**
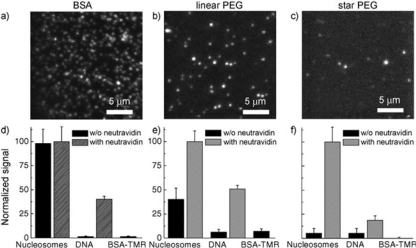
**Upper:** Images illustrating the non-specific binding on labeled nucleosomes on coverslips coated with BSA (**a**), PEG (**b**) and star-PEG (**c**). **Lower:** Quantification based on the number of fluorescent spots. Image used with permission from [126].

A more common method to avoid non-specific interactions, is to pre-coat the surface with strongly adhering proteins. Subsequently, the resulting formed thin protein layer blocks in turn the surface recognition by other molecules. A typical protein of this type is bovine albumin (BSA) [134], which can be biotinylated, providing thus the possibility of a biotin-streptavidin linkage (Figure 5a). This technique is rather straightforward to implement and can be performed in situ, once the microfluidics or flow channels have been realized. However, it has been shown that the technique does not exhibit the highest level of non-specific adsorption prevention [126] and to also potentially interfere with the kinetics of immobilized molecules, such as ribozymes [127,135] (Figure 6).

An alternative approach for in-situ passivation of microchannels was recently demonstrated by Illumina in single molecule sequencing [136]. The technique involved the polymerization of acrylamide in glass capillaries and exhibited significant prevention against non-specific recognition, high sensitivity in isolated nucleic acid imaging, as well as stability over repeated polymerase chain reactions (PCR) [128]. With regards to single molecule DNA sequencing, another technique is based on the use of polyelectrolyte films (Figure 5b). One report focused on the use of single polyacrilic acid layers and was shown to exhibit low tendency to attract fluorescently labeled bases [137]. Multiple films can also be used [15,138,140]. Polyelectrolyte multi-layer films effectively tune the charge density of the substrate and thus exhibit the capacity to repel molecules with the same charge via electrostatic interactions.

Similar strategies of in-situ passivation of PDMS microchannels involve the use of the Pluronic copolymer [141,142]. However, most Pluronic treatment applications focus on the use of microfluidics as cell-culture systems. As an alternative, individual components of the Pluronic block-copolymers, namely poly(ethylene oxide) grafted on oxide surfaces, have been extensively studied in single molecule imaging. Such passivation treatments were shown to exhibit near zero adsorption both when the polymer exhibits linear and star-shape form and exhibit bioactivity evidenced by the reversible denaturation and renaturation of immobilized ribonucleases H (RNase H) [143,144]. However, grafting of PEO requires the surface treatment with aminosilanes, which can exhibit certain disadvantages for in-situ passivation, especially when used with PDMS which swells under treatment with organic solvents [145].

Supported lipid bilayers have also been extensively explored as protein resistant surfaces for single molecule studies (Figure 5c) [146]. In addition to their bio-fouling properties, bilayers exhibit a chemical nature that simulates that of the cellular environment and as a result specifically immobilized biomolecules are expected to maintain their bioactivity [146]. Such membranes are commonly spontaneously assembled from small unilamellar vesicles [147]. Once formed, these bilayers behave like true two-dimensional fluids and any molecules residing on them diffuse freely in the surface plane [148]. Such architectures have found substantial applications within the area of DNA manipulation and stretching by flow [149,150]. In these experiments, highly ordered DNA arrays are formed at localized microscale lipid diffusion barriers, forming thus an elegant assay for highly multiplexed nucleic acid structural studies [151]. On the contrary, dehydration, e.g. occurring during molecular combing, may be considered a disadvantage of this method.

**Figure 7.**
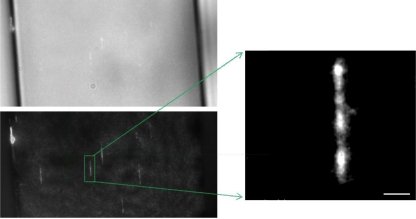
**Left:** Microscopy images of combed λ-phage DNA molecules on a PLL-*g*-PEG coated microfluidic surfaces; the upper and bottom images are under white light and fluorescent conditions respectively. **Right:** An optically magnified image of an individual nucleic acid; the scale bar is 1 μm. In these experiments, the copolymer aided both the prevention of non-specific interactions, but also the stretching of the DNA below its contour length due to the hydrophilic nature of the PEG [40].

The final surface functionalization approach we will discuss and possibly the most common one is poly(ethylene glycol) (PEG) (Figure 5d). PEG has been characterized as the benchmark in resistance of non-specific interactions via steric hindrance and has found a plethora of applications in implants, cell cultures, microfluidics and protein circulation in vivo [152–156]. PEG however does not react with untreated surfaces, thus necessitating an additional surface treatment step. One method is to perform the functionalization ex-situ and then fabricate the microfluidic channels [157]; however, many microfluidic architectures, especially lithographically defined ones, are not compatible with this method as they require the invasive step of bonding and sealing. As a result, several methods have been developed to address this and perform in-situ PEGylation of microfluidic surfaces. Physisorption with PEG copolymers is one of the simplest ones, and involves only the incubation of aqueous buffers of the copolymer. Poly(l-lysine)-*g*-poly(ethylene glycol) (PLL-*g*-PEG) is one such copolymer, which has found numerous applications in both oxide and PDMS based microfluidics for cell cultures, protein studies and single molecule manipulation and imaging [40,153,158,159] (Figure 7). An alternative PEG surface functionalization is based on the covalent bonding of PEG to albumin microgels, which are recognized by the surface [160]. This technique however remains to be fully explored in single molecule imaging applications. Despite recent efforts, little has been achieved specifically in replacing PEG. One such example is dextran [161], which however requires additional efforts to render the functionalization process less toxic [162].

Alternative means for in situ PEGylation of microfluidic surfaces involves direct grafting, usually by first treating the microfluidic surface and subsequently introducing the PEG solution. These processes are usually laborious and require long surface treatments that can interfere with the stability of the microchannels; however they have been shown to provide uniform and extremely stable functionalized surfaces [163–165]. Star-PEG has been used with similar methods, and shown to exhibit superior protein adsorption resistance due to its higher surface coverage and thickness (Figure 6) [126,166]. Alternative techniques of functionalizing surfaces with PEG involve directly mixing the PDMS prepolymer with PEG additives terminated with appropriate end groups [167,168], or by photografting [169,170], which also poses an elegant method for *in-situ* creating chemical patterns. Finally, the direct imprinting of PEG films with microchannels has been reported with some excellent results on non-biofouling, albeit with reported challenges in bonding and sealing [171,172].

## 5. Conclusions

We have reviewed a wide range of methods for enhancing the signal to noise ratio of single molecule imaging in microfluidics. Photonic structures can be directly integrated in the vicinity of micro-channels, forming thus optofluidic platforms that both minimize the background and enhance the signal. Multiple such structures exist that can be adopted for different fluorophores and types of measurements. When choosing, care needs to be taken in order not to substantially modify the photophysical properties of the labels, or if desired, to do it in a controlled way. Due to their fabrication simplicity (bottom-up mostly), plasmonic resonators have been explored more than dielectric ones for single molecule detection; however, recent reports demonstrate the possibility of achieving comparable performances.

With regards to microfluidic approaches, we reviewed architectures that can enhance the sensitivity in single molecule imaging. The latter was mostly related to time domain resolution, stability, and volume restriction methods for adapting the confocal principle in microfluidics. Finally, common surface passivation methods were reviewed; in general, there is no ideal procedure applicable to all types of single molecule experiments. We have attempted to highlight the most popular techniques that are synergetic to microfluidics, both in terms of fabrication and simplicity. One exciting emerging concept to this end is the aptamer surface functionalization [173]; however, apart from affinity extractions and separations, little has been reported within the context of single molecule imaging.
